# Assessing the impact of piercing-sucking pests on greenhouse-grown industrial hemp (*Cannabis sativa* L*.*)

**DOI:** 10.1093/ee/nvad044

**Published:** 2023-05-08

**Authors:** Melissa Pulkoski, Hannah Burrack

**Affiliations:** Department of Entomology and Plant Pathology, North Carolina State University, Raleigh, NC 27695, USA; Department of Entomology, Michigan State University, East Lansing, MI 48824, USA

**Keywords:** hemp, piercing-sucking, pests, greenhouse

## Abstract

*Cannabis sativa* or hemp, defined as <0.3% total tetrahydrocannabinol (THC), is a specialty crop in the United States, of particular interest among growers in the southeastern United States to replace tobacco production. *Tetranychus urticae* (twospotted spider mite), *Aculops cannabicola* (hemp russet mite), *Polyphagotarsonemus latus* (broad mites), and *Phorodon cannabis* (cannabis aphids) are considered the most significant pests in greenhouse grown hemp. Mite and aphid injury can cause cupping and yellowing of leaves, resulting in leaf drop, and reduced flower and resin production. We sought to understand the effects of feeding by *T. urticae* and *Myzus persicae* (green peach aphid), as a proxy for *P. cannabis*, on the concentration of economically significant cannabinoids through a series of experiments on greenhouse grown plants. First, we compared the variability of chemical concentrations in samples collected from individual plants versus pooled samples from 5 plants, and found that chemical concentrations in single plants were similar to those in pooled plant samples. Next, we compared chemical concentrations prior to arthropod infestation and post infestation. When evaluating the mite feeding damage in 2020, cannabinoids in plants infested with high densities of *T. urticae* increased more slowly than in uninfested control plants or plants infested with low *T. urticae* densities. In 2021, the concentration of tetrahydrocannabinol did not differ significantly between treatments. Cannabidiol increased more slowly in plants with low *T. urticae* densities when compared to uninfested controls but did not differ from the high *T. urticae* densities 14 days after infestation.

## Introduction

In the early 20th century, the production of hemp, *Cannabis sativa* L., was essentially banned in the United States following the passing of restrictive laws and heavy taxes (Addlesperger 2015). Until this prohibition, hemp was a staple commodity used for food, fuel, fiber, medicine, clothing, and shelter (Clarke 1999, McPartland et al. 2019). Interest in reintroducing hemp gained traction in the early 21st century, leading to the inclusion of pilot hemp research programs in the 2014 Farm Bill and the removal of hemp and hemp seeds from the DEA schedule of controlled substances in the 2018 Farm Bill. The 2014 Farm Bill allowed for the reentry of hemp into US agriculture by allowing states to create pilot programs as well as protecting and supporting research into the production system (U.S. H.R. 2642 – 113th Congress [113–333]). The 2018 Farm Bill defined hemp as any part of *C. sativa* L. containing less than 0.3% tetrahydrocannabinol (THC) by dry weight (U.S. H.R. 2 –115th Congress [115-334]). This categorized hemp as a distinctly different crop from “marijuana” (high-THC *C. sativa*) and removed it from the federal list of controlled substances. With this deregulation in the United States, hemp is now a growing agricultural specialty crop grown for three primary markets: seed, fiber, and floral production. Floral production, where female plants are grown to produce unfertilized flower which are either used to extract cannabinoids or dried for use as a smokable product, was the initial interest for many new growers exploring hemp because it was thought to have the potential to be a high value crop (Schluttenhofer and Yuan 2017, Jelliffe et al. 2020). Immediately following the 2018 Farm Bill, there was significant interest in hemp production in North Carolina, where we conducted our research. A survey of organically certified growers indicated that nearly 85% were open to trying hemp production on their farm, although this interest was contingent on many other factors included the willingness of organic certifiers to approve hemp (97%), markets existing for hemp products (94%), and most interesting for our work, low insect pressure (94%) (Dingha et al. 2019). Field hemp production in North Carolina increased from 781 ha (1,930 acres) in 2017 to 4,683 ha (11,572 acres) in 2019 (Mark et al. 2020) with 5,690 licensed ha (14,016 acres) reported in 2021, the last year for which data were provided by the state level industrial hemp commission (North Carolina Industrial Hemp Commission 2021).

Most hemp production practices were lost during prohibition, essentially making it a new agricultural crop in the United States. In the Southeast, there has been interest in utilizing hemp to replace lost tobacco production and revenue and to diversify crop rotations, but the gap in production knowledge has raised questions about fertility management, field layout, and disease and insect management. In 2021, US growers planted 21,915 open hectares (13,549 harvested hectares) for a production value of $712 million (USDA NASS 2022). Hemp can also be grown to maturity indoors, and in 2021, there were 1,449,287 square meters of greenhouse hemp production with a production value of $112 million.

Hemp plants are produced in two ways, either from seeds started in seed trays or asexually propagated clones cut from a mother plant. For floral or cannabinoid production, plants grown either from seeds or as clones are maintained in the greenhouse as future mother plants, for production in the greenhouse, or until they are large enough to transplant into the field. Arthropod pests can attack plants at all stages of production (Cranshaw et al. 2019), and our work has focused on potential pest impact within the greenhouse.

**Fig. 1.**
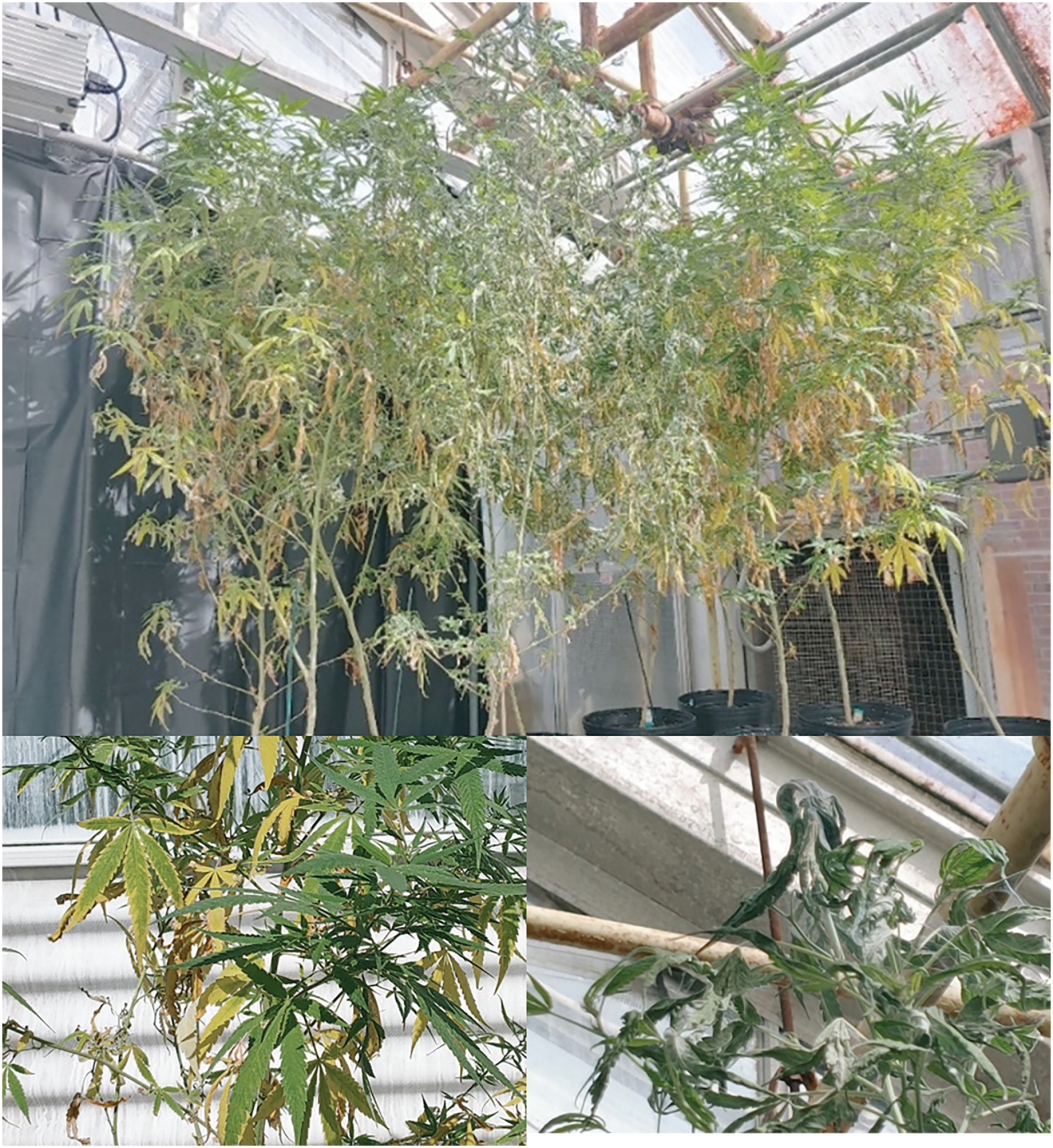
Injury due to twospotted spider mite feeding and webbing. (Photographs taken by Melissa Pulkoski).

A survey of growers in 2021 reported that 5% of respondents lost acreage due to testing above the 0.3% THC legal limit, a loss that is predicted to increase now that state pilot programs have ended, and 75% lost acreage due to various pests, up from 40% in 2020 (Hemp Benchmarks 2021). This sharp increase in loss may be at least in part to increased awareness of pest damage. Hemp russet mites (*Aculops cannibicola*), twospotted spider mites (*Tetranychus urticae*, TSSM), and broad mites (*Polyphagotarsonemus latus*), feeding on mesophyll tissue, and cannabis aphids (*Phorodon cannabis*), feeding on phloem, are considered the top greenhouse hemp pests (Cranshaw et al. 2019). Mite and aphid injury initially results in leaf drop, and the latter reduces flower and resin production, eventually killing plants (personal observation, Fig. 1). Growers report fewer issues with mite pests in the field, but they cause significant damage in the greenhouse, and it is critical to develop sustainable pest control strategies to protect mothers, seedlings, and clones prior to transplant (Cranshaw et al. 2019). Currently, there are few pesticides registered for use in hemp (Visković et al. 2023), none with proven efficacy, and there are limited research-based recommendations for insect or mite management in hemp (Britt and Kuhar 2020).

When damaged by insects, plants can modify their chemical production. Plants such as tomatoes, cotton, and wild and cultivated tobacco have evolved these changes in chemical production as resistance mechanisms to reduce injury and yield loss (Mitchell et al. 2016). These resistance mechanisms include 2 types of defensive chemicals: constitutive and induced. Constitutive defenses are always present and can be used for reproduction, to create physical barriers, and provide structure and stability to the plant (Freeman and Beattie 2008). Induced resistance mechanisms are upregulated following damage by insect pests. These can include physical defenses such as trichomes (Yoshida et al. 2009). Non-glandular trichomes create a physical barrier that can prevent insects from reaching the leaf surface preventing feeding or oviposition, and can also impale soft bodied insects (i.e., mites and aphids) while glandular trichomes secrete toxic chemicals and oils to deter or repel insects (Leven 1973). Inducible defenses are triggered by an outside force, such as mite feeding triggering the salicylic acid, jasmonate, or ethylene pathways (Arena et al. 2016). When tomatoes (*Solanum lycopersicum*) and lima beans (*Phaseolus lunatus*) are infested by spider mites, these pathways release volatile organic compounds that attract predators (De Boer et al. 2014). In cotton plants, terpenoid aldehydes, such as gossypol found in the pigment glands, increase when fed on by arthropod pests. The increase of gossypol negatively impacts several lepidopteran herbivores; however, mites seem unaffected (Agrawal and Karban 2000). Strawberry (*Fragaria x ananassa (Weston) Duchesne ex Rozier (pro sp.)*) plants produce oxidative enzymes to prevent feeding when attacked by mites (Steinite and Ievinsh 2003).

Chemicals in hemp, such as THC and other cannabinoids, are presumed to be related to plant defense against environmental stress, including insect and mite feeding (Sirikantaramas et al. 2005, Bidart-Bouzat and Imeh-Nathaniel 2008). Cannabiniod concentrations in *C. sativa* have been documented to increase under nutrient, drought, and UV stress and in some cases under temperature stress (Gorelick and Bernstein 2017). The effects of biotic stressor on cannabis have been less studied. Cannbinoids have been reported to increase in plants that have been chewed (McPartland et al. 2000), and cannbiniods have been documented to elicit behavorial changes in insects (Rothschild and Fairbairn 1980). Interestingly, insects appear to lack cannabinoid receptors, and it has been proposed that changes in insect behavior caused by cannabis or cannabinoids may mediated by other chemical pathways (McPartland et al. 2001). We are not aware of similar assessment of the presence of cannabinoid receptors in Acari. Because of the known effects of arthropod feeding on induced plant responses in other systems, growers are concerned about pest damage effects on the chemical concentrations in hemp (Cranshaw et al. 2019). The concentration of THC is important because hemp must contain less than 0.3% THC, and the concentration of cannabidiol (CBD) is important to hemp growers seeking to extract this compound for sale.

Due to the evidence of arthropod feeding affects on secondary plant chemicals in other cropping systems, we hypothesized that biotic stress due to insect damage may increase the concentration of THC and other cannabinoids in hemp. We further expected the plant response may differ due to the feeding modalities between spider mites, aphids, and caterpillars (see Rodriguez-Saona et al 2005 for an example). We conducted a series of experiments on greenhouse grown hemp plants to evaluate the impact of key arthropod pests on total THC and CBD concentrations. First, we determined if flower samples collected from single plants or pooled flower samples from multiple plants most accurately represented THC and CBD concentration measurements in order to inform future experiments. Then, we conducted experiments aimed to evaluate the effect of spider mite and aphid feeding damage on hemp.

## Materials and Methods

### Preparation of Plant Materials

All experiments were conducted at the Method Road Greenhouses located on the North Carolina State University campus (Raleigh, NC), and plant growing procedures were the same for all studies. Five-week-old asexually propagated var. BaOx clones were used for experiments in 2020 and 2021, (Carolina Greenhouses, Kinston, NC) and were transplanted into 3-gallon black pots filled with Sungro professional growing mix (Sun Gro Horticulture, Agawam, MA). Grow mix consisted of 75–85% Canadian sphagnum peat moss, perlite, dolomite limestone, and wetting agents. Plants were fertilized bi-weekly at a rate of 124.8 mg/L with Jack’s Professional Peters General Purpose 20-10-20 fertilizer (JR Peters, Inc., Allentown, PA). Plants were hand watered every 2 to 3 days as needed to prevent wilting. Greenhouse lights were set to an 18-hour photoperiod during an eight-week vegetative growth period at an average temp of 27˚C. Lights were then switched to a 12-hour photoperiod to induce flowering. To ensure the correct photoperiods were achieved, greenhouse walls were covered with 10 mm black plastic to exclude light. Seed for a proprietary auto flowering variety, referred to hereafter as var. Autoflower, was donated by a cooperating grower for use in an additional experiment in 2021. Var. Autoflower plants were grown from seed, and ambient light conditions were maintained for the entire growing period because auto flower plants are not sensitive to photoperiod (McPartland 2018). Other plant growth conditions were the same as previous experiments. Treatments were arranged in a randomized complete block design (RCBD) for all experiments.

### Floral Sample Collection

Pre-infestation floral samples were collected at 17 weeks after transplant for BaOx plants in 2020 experiments, 9 weeks after transplant for BaOx planted in 2021 experiments, and 11 weeks after seeding for Autoflower plants used in 2021 experiments. Floral samples in all experiments were collected by removing approximately 3 g of randomly selected flower material from each replicate using Fiskars Stainless Steel Garden Snips (Fiskars, Finland). Samples were immediately packaged in paper bags, to prevent moisture collection, and delivered to Delta 9 Analytical (Raleigh, NC) for analysis via high performance liquid chromatography (LC-20 HPLC System with CTC PAL HTS Autosampler, Shimadzu, Japan) and gas chromatography mass spectrometry (Sciex API 4000 Mass Spectrometer). Samples were air-dried, ground, and homogenized. Approximately 50 mg subsamples were identified for chemical analysis. Subsamples were analyzed for Cannabinol (CBN), Delta 8 tetrahydrocannabinol (THC), Cannabichromene (CBC), Cannabigerol (CBG), Cannabidiol (CBD), Cannabigerolic Acid (CBGA), Cannaabidivarin (CBDV), Cannabidiolic Acid (CBDA), Delta 9 Tetrahydro-cannabinolic Acid (THCA), Tetrahydrocannabidivarin (THCV), Delta 9 THC, Total THC, and Total CBD (not all chemistries were detected). Total THC and CBD concentration were calculated as follows: THC = THCA × 0.877 + Delta 9 THC and CBD = CBDA × 0.877 + CBD (Stack et al. 2021).

### Sampling Method Development

**Fig. 2.**
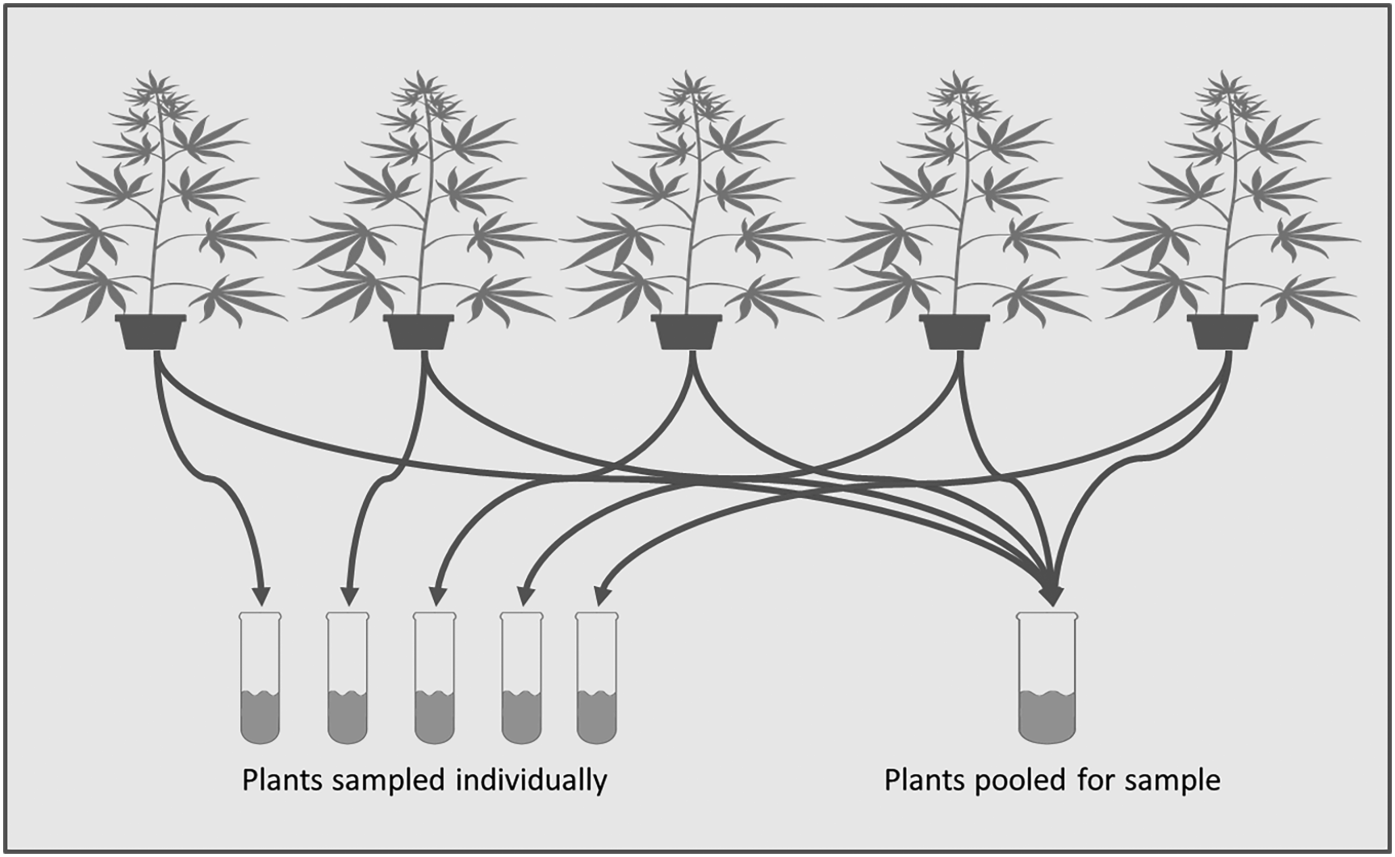
Each plant in the first replicate was sampled individually, and those same plants were then pooled into a single sample. Pooled samples were collected from the remaining 3 replicates.

A greenhouse experiment was conducted in 2020 to evaluate if pooled samples from several plants within a replicate accurately represented the average of the individual plants for the chemical concentrations of total THC and CBD in hemp. Plants were assigned to 4 replicates, each containing 5 plants and grown under the conditions listed above. Three weeks after flower initiation, samples were collected from 5 plants individually, and a separate pooled sample was collected from the same 5 plants (Fig. 2) as well as 3 other replicates of 5 plants. A second set of samples were taken at either 7, 10, or 14 days later (replicates were pulled at different times due to changes in plant health among treatments).

### *Tetranychus urticae* Feeding Damage

Three experiments aimed to evaluate the effect of *T. urticae* feeding on hemp chemistry and were conducted in the summer of 2020 (var. BaOx), summer 2021 (var. BaOx), and winter of 2021 (var. Autoflower). Two weeks prior to *T. urticae* infestation, greenhouse grown plants were treated with imidacloprid (equivalent to 82.8 ml/ha at 468 L/ha volume of Admire Pro, Bayer Crop Sciences, Research Triangle Park, NC) to prevent the establishment of non-target pests. Control plants were treated with fenpyroximate (equivalent to 1,705 ml/ha at 189 L/ha volume Portal Miticide, Nichino American, Wilmington, DE) one day prior to infestation of mite treatments and remained mite throughout experiments.

In 2020, 3 treatments of 5 single plant replicates were compared. Three weeks after flower initiation, pre-infestation samples were taken and then plants were artificially infested with *T. urticae*. Treatments included an un-infested control, low *T. urticae* infestation rate (~50 mites per plant), and high *T. urticae* infestation rate (~200 mites per plant). Due to the rapid establishment of mites and poor plant health, terminal samples were taken for the high infestation rate after 7 days. Terminal samples were taken for the low infestation rate at 10 days after infestation. In addition, 5 leaves were collected from the middle and upper third of plants in each replicate at the same time as floral samples to evaluate mite density. Leaves were observed under a stereomicroscope between 10× to 60× magnification, and motile mites and mite eggs were enumerated.

During the summer of 2021, plants were assigned to 3 treatments of 10 single plant replicates. Because of the rapid plant decline observed in the first experiment, lower infestation rates were evaluated: an uninfested control, low *T. urticae* infestation (~25 mites per plant), and high *T. urticae* infestation (~125 mites per plant). Pre-infestation and 4, 7, 11, and 14 days after infestation samples were collected and handled as described above. Three leaves were collected from the middle and upper third of each plant at each sample point to evaluate the average motile mites using a stereomicroscope.

During the winter of 2021, var. Autoflower plants were assigned to 2 treatments of 10 single plant replicates: control and *T. urticae* infested (~25 per plant). This lower density was selected because autoflower plants are approximately one fifth the size of the var. BaOx plants used in prior experiments. Pre-infestation, 7 and 14 days after infestation samples were collected and handled as described above. Three leaves were collected from each plant to evaluate the average motile mites using a stereomicroscope.

### Aphid Suitability

A series of experiments was also conducted to determine if *Myzus persicae* (green peach aphids) can survive and reproduce on whole plant vegetative hemp. Previous laboratory bioassays conducted in the summer of 2019 suggested that *M. persicae* from a laboratory colony reared on tobacco could survive and reproduce on excised hemp leaf clippings (Burrack, unpublished data). However, when we attempted to infest whole plants to assess chemical effects, this species did not establish. Two experiments were conducted comparing the ability of *M. persicae* to survive and reproduce on whole plants, excised leaf clippings, and leaf disks.

We first compared 3 treatments with 10 replications each. Fifteen leaves were collected from greenhouse-grown var. BaOx plants and used to conduct 2 laboratory bioassays, and 10 whole plants were used for whole plant evaluation. Prior to leaf collection plants were treated with fenpyroximate (equivalent to 3.5 L/ha at 935 L/ha volume of Portal, Nichino America, Wilmington, DE) to prevent mite establishment. Ten whole leaves were embedded in a Petri dish with a dilute agar solution (4 g agar/800 ml water) as it cooled but before it solidified. The remaining 5 leaves were cut, using a 2.5-cm leaf punch, to fit in 30 ml cups, and embedded in dilute agar. A single leaf on each whole plant was identified and marked with embroidery thread. Five adult aphids from a laboratory colony reared on tobacco, were placed in each bioassay arena or on a plant. Bioassay arenas were held upside down on trays in the laboratory under ambient light and temperature conditions. Greenhouse plants were held under same greenhouse conditions as listed above and aphids were allowed to feed and reproduce for up to 7 days. Arenas and plants were evaluated 3 and 7 days after infestation.

A second experiment was conducted using the same method but with 2 aphid populations, one which had been reared in the laboratory on excised hemp leaf clippings for 2 reproductive cycles, and the original population reared on tobacco leaves. To start and maintain the laboratory hemp colony either 3 adult aphids or 5 nymphs were placed in plastic 1 oz cups that had a one-inch hemp leaf disc set into a 1% agar solution. The cups were maintained upside down in a growth chamber at 20˚C under a 12-hour photoperiod and changed weekly.

### Statistical Analysis

Sampling method data were analyzed via a mixed model analysis of variance fit to a log distribution PROC GLIMMIX (SAS v9.4, Cary, NC) with days after infestation, treatment, sample method (pooled or single plant), and the interaction between treatment and sample method included as fixed effects. Replicate was considered a random effect. Because single plant samples were collected from each of 5 plants in one replicate, that replicate was excluded from pooled samples, meaning there were 5 single plant and 3 pool replicates. The means and associated 95% confidence intervals for sampling method data were also calculated via PROC MEANS (SAS, v.9.4, Cary, NC) and compared to determine if sampling methods were likely to produce different results.

Chemical data from mite infested plants were analyzed 2 ways. First, we subtracted the final THC or CBD value for a replicate from the pre-infestation value, here after referred to as “change over time”. We also compared the absolute values at each observed time point. The 2020 BaOx mite feeding data were compared via a mixed model analysis of variance fit to a log distribution to meet the assumptions of ANOVA with replicate as a random effect and treatment as a fixed effect (PROC GLIMMIX, SAS, v.9.4, Cary, NC). The 2021 BaOx and Autoflower mite feeding data met the assumptions of ANOVA and were analyzed via a mixed model analysis of variance with replicate as a random effect and treatment, days after infestation, and their interaction as fixed effects (PROC MIXED, SAS, v.9.4, Cary, NC). Linear regression analyses (Proc GLM, SAS, v.9.4, Cary, NC) were conducted to determine the relationship between *T. urticae* densities on leaves and chemical concentrations within the same plant. Treatment and days after infestation were included as fixed effects in the model.

Data from the first aphid experiment with tobacco only reared aphids were analyzed via a PROC GLIMMIX with a log distribution with replicate as a random effect and arena (plant, whole leaf, and leaf disc), days after infestation, and their interaction as fixed effects. Data from the second aphid experiment with hemp and tobacco reared aphids were analyzed via a PROC GLIMMIX with a log distribution with replicate as a random effect and colony source (hemp or tobacco), arena, days after infestation, and their interactions as fixed effects. In instances where significant effects were observed, adjusted means were separated using the Tukey–Kramer adjustment (α = 0.05).

## Results

### Sampling Method Development

**Fig. 3.**
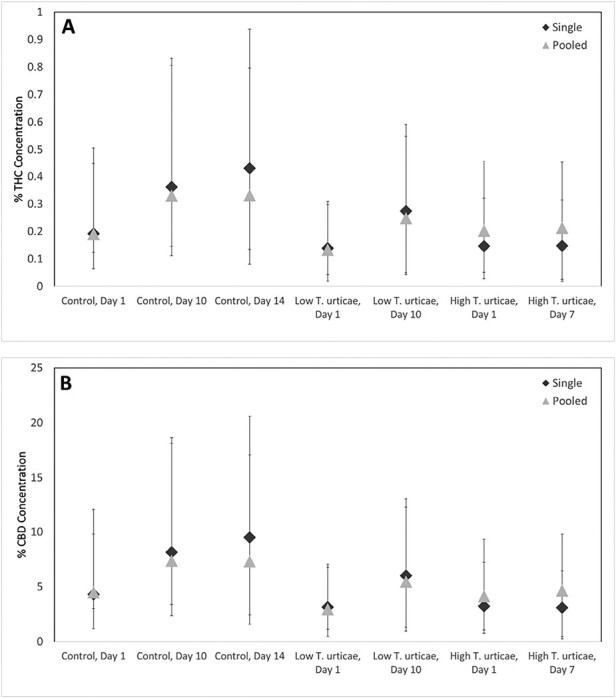
Mean percentage concentration by dry weight ± 95% confidence interval for the individual samples (diamonds) for both THC A) and CBD B) as compared to values for pooled samples ± 95% confidence interval (triangles).

There was no significant effect of sampling method on the concentration of either chemical (THC: *F* = 0.53; df = 1,55; *P* = 0.4700; CBD:*F* = 0.62; df = 1,55; *P* = 0.4336). In addition, means for pooled samples fell within the 95% CI for THC and CBD, and results of the single plant analyses were similar to pooled plot analyses (Fig. 3). Subsequent experiments were conducted on single plant replicates.

### *Tetranychus urticae* Feeding Damage

**Fig. 4.**
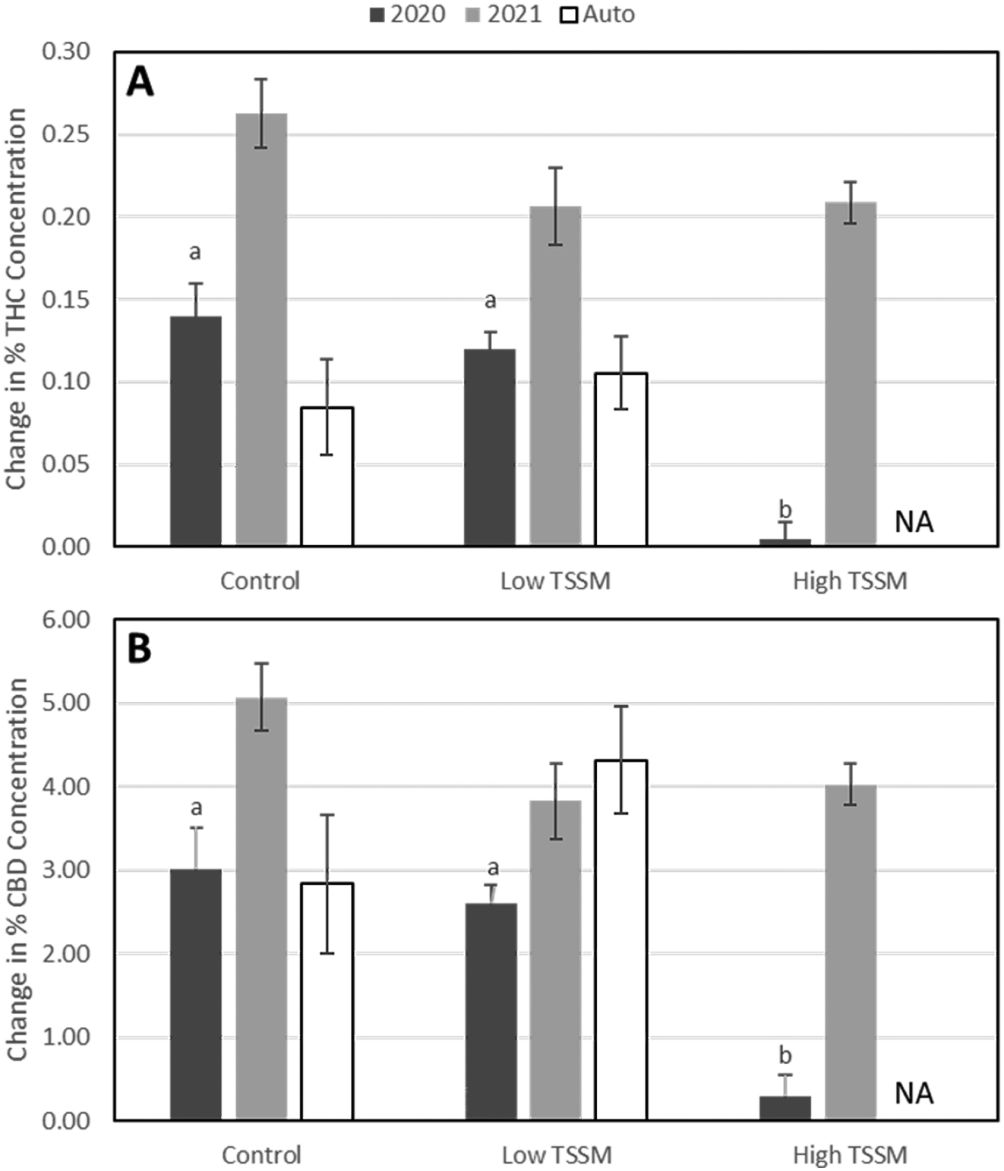
Mean ± SEM change in percentage concentration (final concentration − initial concentration) by dry weight for THC A) and CBD B) over time by treatment (uninfested, Low TSSM, and High TSSM) and experiment (2020 BaOx, 2021 BaOx, and 2021 Autoflower). Means indicated by the same letter within a given chemical are not significantly different, = 0.05, via the Tukey–Kramer adjustment. High TSSM treatment was dropped for Autoflower.

In 2020, THC and CBD change over time at high levels of *T. urticae* infestation was significantly lower than uninfested plants or those with lower *T. urticae* densities (THC: *F* = 44.40; df = 2,6; *P* = 0.0003; CBD: *F* = 19.18; df = 2,6; *P* = 0.0025) (Fig. 4). In 2021, THC and CBD concentrations in high and low *T. urticae* infested plants experienced the same change over time as the uninfested control (THC: *F* = 2.71; df = 2,18; *P* = 0.0934. CBD: *F* = 3.08; df *=* 2,18; *P =* 0.0705) (Fig. 4).

**Fig. 5.**
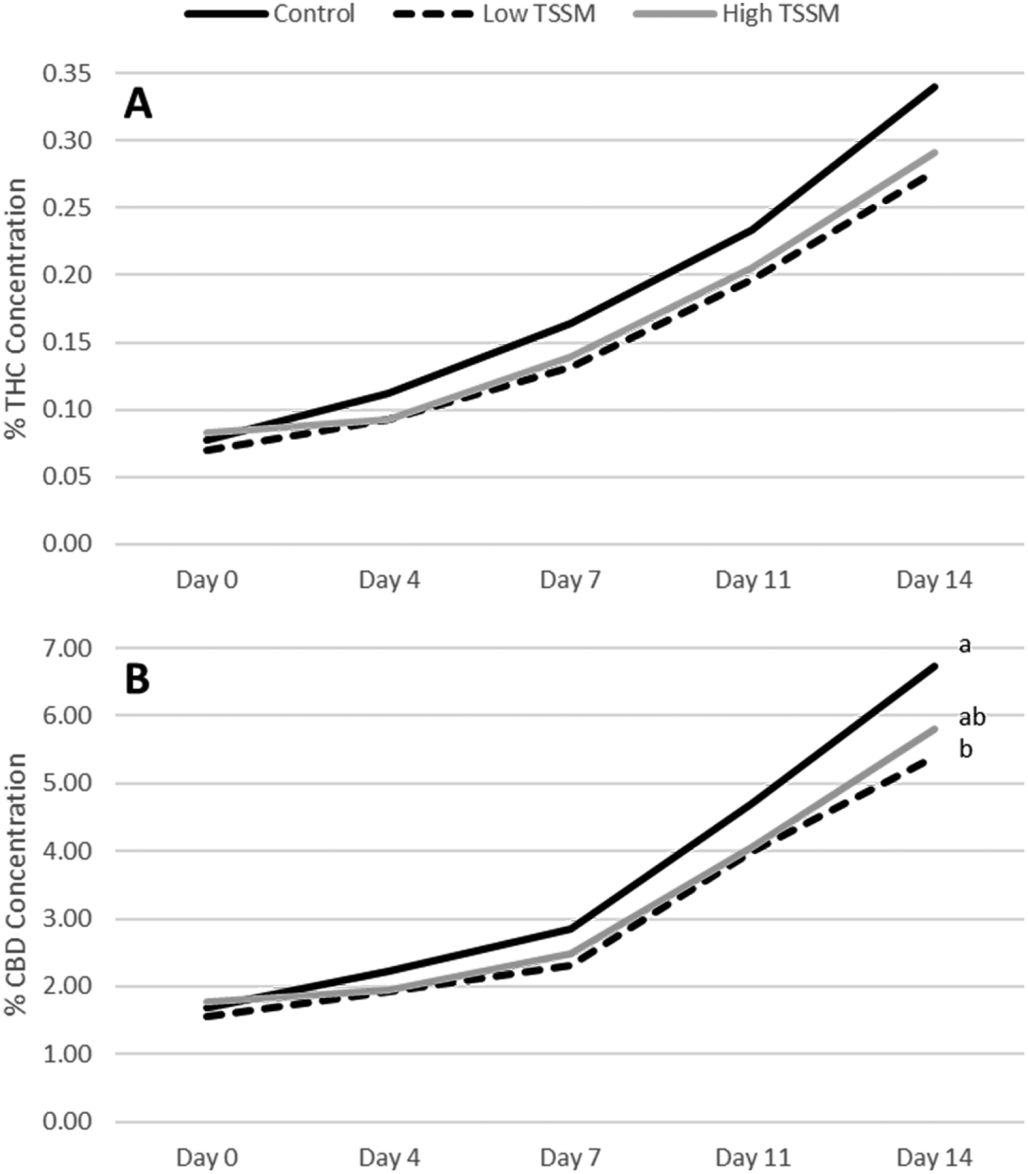
In 2021, the change in percentage concentration by dry weight for THC A) did not significantly differ over time following *T. urticae* infestation. The rate of CBD increase B) did not differ on the first 3 sample points but was significantly lower at low *T. urticae* densities than the control 14 days after infestation.

However, at 14 days after infestation, plants infested with the lower rate of *T. urticae* had significantly lower CBD concentrations than uninfested plants, while the higher infestation rate was not significantly different from the control or the lower infestation rate (CBD: *F =* 3.25; df *=* 2,27; *P =* 0.0487) (Fig. 5). Mite infestation did not significantly impact THC or CBD concentration in var. Autoflower (THC: *F =* 0.34; df *=* 1,9; *P =* 0.5766. CBD: *F* = 2.08; df = 1,9; *P =* 0.1828) (Fig. 5).

**Fig. 6.**
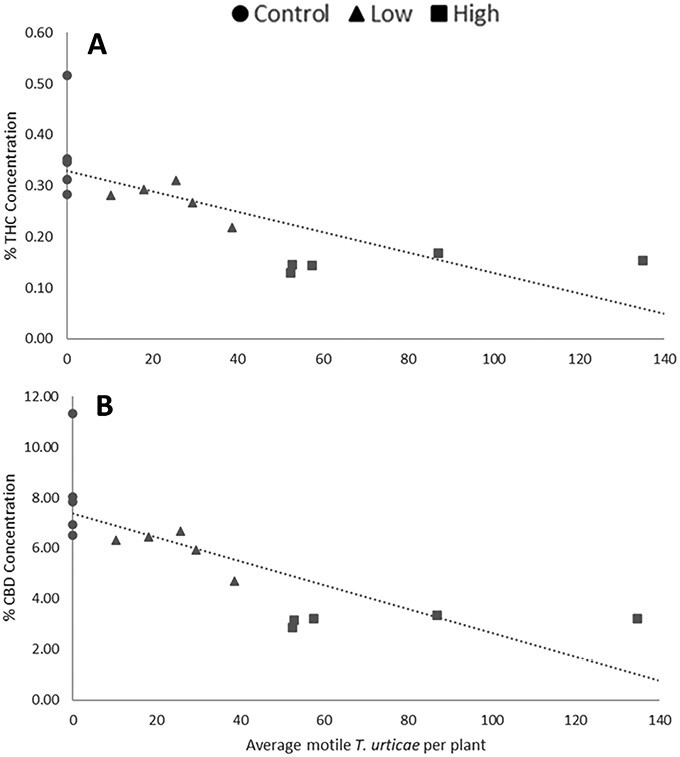
In 2020, higher densities of motile mites were negatively correlated with both THC A) and CBD B); (THC: *y* = −0.0020*x* + 0.3292, CBD: *y* = −0.0471*x* + 7.3649).

**Fig. 7.**
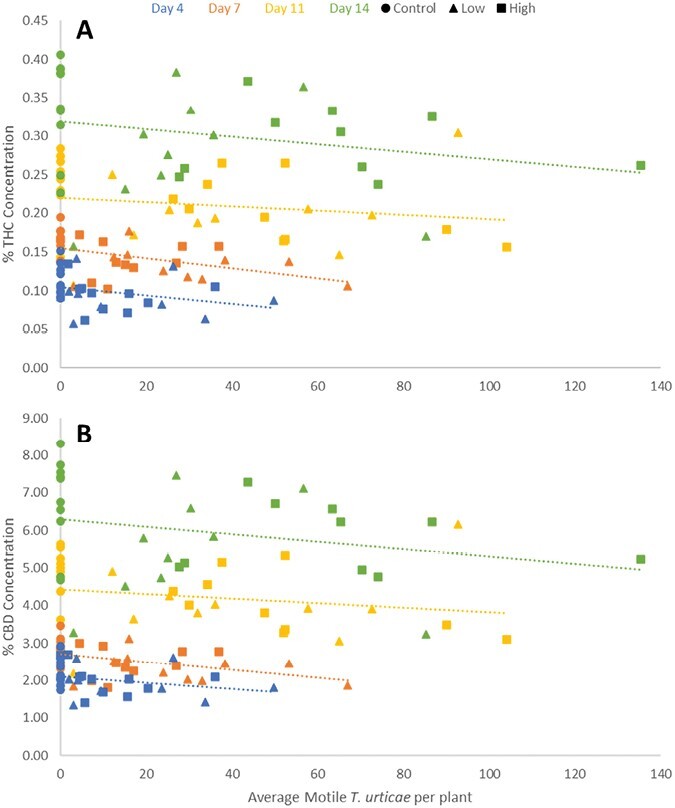
Higher densities of motile mites are negatively correlated with both THC A) and CBD B) in 2021 experiments.

Linear regressions of mite counts and cannabinoid concentration from the same plant in 2020 var. BaOx revealed significant increase over time for THC (DAI: *F* = 9; df = 1,11; *P =* 0.0121) and a significant negative interaction between mite concentration and time effect for CBD (mites × DAI: *F* = 6.34; df = 1,11; *P =* 0.0286) (Fig. 6). In 2021 experiments on var. BaOx, THC and CBD decreased in response to mite concentration (THC: *F* = 5.29; df = 1,112; *P =* 0.0232. CBD: *F* = 4.47; df = 1,112; *P =* 0.0366) and increased over time (THC: *F* = 80.05; df = 3,112; *P* < 0.0001. CBD: *F* = 85; df = 3,112; *P* < 0.0001)(Fig. 7). Mite numbers were not significantly related to THC or CBD in var. Autoflower when assessed in 2021.

### Aphid Suitability

In our initial experiments, no tobacco-reared *M. persicae* adults survived on hemp plants, and significantly fewer survived on whole leaves as compared to excised leaf segments (Means ± SEM: Whole plant 0.00 ± 0.00, Leaf 0.55 ± 0.21, Leaf Disc 3.65 ± 0.03; *F =* 139.54; df = 2, 45; *P* < 0.0001). Tobacco-reared *M. persicae* nymph production was greater on excised leaf discs and remained the same on whole leaves; no nymphs were produced by tobacco reared adults placed on whole hemp plants (Experiment 1, Table 1, Arena × DAI: *F =* 4.31; df = 2, 45; *P =* 0.0195).

**Table 1.** Mean *Myzus persicae* nymph production ± SEM on excised hemp leaf discs, whole leaves, and plants

	Experiment 1		Experiment 2			
	Tobacco-reared		Tobacco-reared		Hemp-reared	
	3 DAI	7 DAI	3 DAI	7 DAI	3 DAI	7 DAI
Leaf disc	9.10 ± 2.29b	27.30 ± 4.69a	14.20 ± 2.78b	18.50 ± 1.75ab	33.30 ± 4.78a	22.30 ± 5.19ab
Whole leaf	2.00 ± 1.79c	1.20 ± 1.20c	0.90 ± 0.60c	1.10 ± 0.77c	0.50 ± 0.34c	0.90 ± 0.55c
Whole plant	0.00 ± 0.00c	0.00 ± 0.00c	0.00 ± 0.00c	0.00 ± 0.00c	0.00 ± 0.00c	0.00 ± 0.00c

Values within an experiment followed by the same letter are not significantly different (α = 0.05) when means were compared using the Tukey–Kramer adjustment.

In the second experiment, the interaction between arena and colony source was not significant (*F* = 2.76; df = 2,99; *P* = 0.0683) for adult *M. persicae*, but more adults survived on leaf discs than on leaves or whole plants (Means ± SEM: Whole plant 0.00 ± 0.00, Leaf 0.20 ± 0.07, Leaf Disc 3.15 ± 0.25; *F* = 179.95; df = 2, 99; *P* < 0.0001), and more adults from the tobacco colony survived than those originating from the hemp-reared colony (Means ± SEM: Hemp-reared 0.95 ± 0.22, Tobacco-reared 1.28 ± 0.23; *F* = 6.27; df = 1, 99; *P =* 0.0139). There was a significant three-way interaction between arena, colony source, and time for nymph production (*F* = 3.83; df = 2,99; *P =* 0.025), but nymph numbers were still highest on excised leaf discs with no nymphs produced on whole hemp plants (Experiment 2, Table 1).

## Discussion

Our results demonstrate that variability for THC and CBD concentrations is similar among individual plants such that means for pooled samples do not differ from those of individual plants. This suggests that experiments with single plants are representative of the variation that would be observed in larger plots and allows researchers to tailor experimental designs. In the case of our observations of the relationship between mite densities, which can vary greatly from plant to plant, and chemistry, single plant observations allow for more closely aligned data and greater replication.

*Tetranychus urticae* has the potential to negatively impact THC and CBD concentration in greenhouse grown hemp, but this effect is time and density dependent and more likely to occur in otherwise stressed plants. Both chemicals increased at a slower rate in comparisons conducted during 2020 on large plants that became more heavily infested, and these differences were observed at 7 and 10 days after infestation. During 2021 experiments on the same variety, differences were not observed in THC concentration and were only observed in CBD concentration at 14 days after infestation.

While cannabis aphids are a known pest in hemp, field populations do not regularly occur in North Carolina, which limited our ability to assess aphid effects (Cranshaw et al. 2019). We attempted to utilize an existing colony of *M. persicae* as a proxy for the known hemp pest *Phorodon cannabis* and were able to determine that it is not likely to be a pest in hemp. We only observed survival and reproduction on leaf discs and were unable to colonize whole plants despite rearing populations on excised leaf discs for 2 reproductive cycles. It is possible that longer term acclimatization may allow *M. persicae* to adapt to feeding on hemp, but this is unlikely to occur in field conditions, as our research showed that nymphs cannot survive on whole plants, likely due to plant defensive chemicals. Leaf disk assays are commonly used to screen for spider mites and aphid resistance on tobacco, lettuce, strawberry, tomato (Ferrer et al. 1993, Kloth et al. 2015, Rakha et al. 2017, Rodriguez et al. 2014). Caution should be taken when applying these methods to hemp, however, given our observations.

We observed that both THC and CBD were negatively affected by *T. urticae* feeding, supporting that they are regulated the similarly within plants. This indicates that with mite feeding there is a tradeoff between plant defense and cannabinoid concentration, rather than an induced increase in these defensive chemicals. Given the legal constraints on THC, this tradeoff could be beneficial to growers interested in grain and fiber varieties but could be a concern for growers interested in producing CBD as mite feeding can also reduce that concentration. Cannabinoid concentrations found in hemp can be difficult to accurately measure due to the small quantities. Several of the chemicals included in mass spectrometry analysis were either not detected or fell below the limit of quantitation and are not reliable (Supplementary Tables 1, 2, and 3). These low concentrations make observing effects and interactions difficult without high replication, especially for cannabinoids such as THC that should remain below 0.3%. However, CBD and CBG concentration tend to be much higher in varieties grown for cannabinoid production (Rupasinghe et al. 2020). It is possible that we were able to observe effects for CBD that would be harder to detect for other chemicals.

Recently published results from a small-scale study conducted in Israel differ from our findings in that cannabinoids increased slightly in *T. urticae* infested plants (Kostanda and Khatib 2022). Plants in this experiment appear to have been infested for a longer duration than ours and were sampled over a 5 month period, although initial *T. urticae* rates were not provided. This group also found that chlorophyll significantly decreased following *T. urticae* infestation which has also been observed in many other crops including strawberries (Sances et al. 1979), peppermint (Deangelis, et al 1983), and cucumber (Park and Lee 2002). Visual observations of yellowed, cupped leaves on plants heavily infested with in *T. urticae* our experiments (Fig. 1) were also consistent with reduced chlorophyll.

This experiment focused on pre-infestation and time points within the following weeks and did not evaluate the effects on cannabinoid concentration at the time of harvest. It would be beneficial to evaluate the long-term effects of mite feeding and effects of constant infestation. Assessing whether cannabinoid concentrations can stabilize if pest pressure is relived prior to harvest, would also be useful, and would be consistent with when growers are required to test by United States Department of Agriculture.

Our work illustrates that further investigation is needed to determine the impact of mite feeding but suggests that unchecked infestations can negative impact cannabinoids in hemp plants grown in the greenhouse. If hemp producers do not take action at the early stages of mite infestation, they could risk both physical injury to plants resulting in yield reduction and also reduction in cannabinoids. Future studies looking into the effects of other pest groups, the effect on plant health in combination with arthropod feeding as well as infestation timing and longer infestations should also be conducted. Determining the impact of pest feeding and timing on cannabinoid profiles will help with developing an effective integrated pest management plan. Knowing which pests could be more economically impactful to THC concentrations in regard to the legal constraint and pesticide application timing are key components in an integrated pest management plan.

## Supplementary Material

nvad044_suppl_Supplementary_Material
