# Supplementary material for: Assessing the impact of piercing-sucking pests on greenhouse-grown industrial hemp (Cannabis sativa L.)
Source: Environ Entomol. 2023 May 8;53(1):1–10. doi: 10.1093/ee/nvad044 (PMC10878361; doi:10.1093/ee/nvad044)
Supplement: nvad044_suppl_Supplementary_Material [file nvad044_suppl_supplementary_material.docx]

SUPPLEMENTARY TABLES

**Supplementary Table 1** Mean ± SEM percentage concentration by dry weight for all tested cannabinoids for 2020 var. BaOx, infested with *T. urticae.* Total THC/CBD concentration used in Chapter 1 were calculated using THC *=* THCA x 0.877 + Delta 9 THC; CBD = CBDA x 0.877 + CBD.

| Cannabinoid | Infestation  Level | Days After Infestation (DAI) | |
| --- | --- | --- | --- |
|  |  | Pre infestation | Post infestation |
| Cannabinol (CBN) | Control  Low  High | ND  ND  ND | ND  ND  ND |
| Delta 8 THC | Control  Low  High | ND  ND  ND | ND  ND  ND |
| Cannabichromene (CBC) | Control  Low  High | ND  ND  ND | ND  ND  ND |
| Cannabigerol (CBG) | Control  Low  High | 0.0148 ± 0.0027  BL  BL | 0.0355 ± 0.0064  0.0139 ± 0.0016  BL |
| Cannabidiol (CBD) | Control  Low  High | 0.0441 ± 0.0047  0.0541 ± 0.0069  0.0501 ± 0.0037 | 0.0819 ± 0.0147  0.0501 ± 0.0027  0.0422 ± 0.0038 |
| Cannabigerolic Acid (CBGA) | Control  Low  High | 0.3491 ± 0.0969  0.1284 ± 0.0464  0.0581 ± 0.0186 | 0.4483 ± 0.0920  0.2666 ± 0.0262  0.0906 ± 0.0193 |
| Cannaabidivarin (CBDV) | Control  Low  High | ND  ND  ND | ND  ND  ND |
| Cannabidiolic Acid (CBDA) | Control  Low  High | 4.8782 ± 0.4732  3.5372 ± 0.1860  3.5260 ± 0.2396 | 9.1832 ± 0.9537  6.8083 ± 0.3936  3.5750 ± 0.0892 |
| Delta 9 Tetrahydro-cannabinolic Acid (THCA) | Control  Low  High | 0.2187 ± 0.0261  0.1588 ± 0.0078  0.1670 ± 0.0117 | 0.4132 ± 0.0461  0.3125 ± 0.0177  0.1693 ± 0.0072 |
| Tetrahydrocannabidivarin (THCV) | Control  Low  High | ND  ND  ND | ND  ND  ND |
| Delta 9 THC | Control  Low  High | BL  BL  BL | BL  BL  BL |

ND = Not detected or no peak present

BL = Below the Limit of Quantitation but above the Limit of Detection

**Supplementary Table 2** Mean ± SEM percentage concentration by dry weight for all tested cannabinoids for 2021 var. BaOx, infested with *T. urticae.* Total THC/CBD concentration used in Chapter 1 were calculated using THC = THCA x 0.877 + Delta 9 THC; CBD = CBDA x 0.877 + CBD.

| Cannabinoid | Infestation  Level | Days After Infestation (DAI) | | | | |
| --- | --- | --- | --- | --- | --- | --- |
|  |  | Pre infestation | 4 DAI | 7 DAI | 11 DAI | 14 DAI |
| Cannabinol (CBN) | Control  Low  High | ND  ND  ND | ND  ND  ND | ND  ND  ND | ND  ND  ND | ND  ND  ND |
| Delta 8 THC | Control  Low  High | ND  ND  ND | ND  ND  ND | ND  ND  ND | ND  ND  ND | ND  ND  ND |
| Cannabichromene (CBC) | Control  Low  High | ND  ND  ND | ND  ND  ND | ND  ND  ND | ND  ND  ND | ND  ND  ND |
| Cannabigerol (CBG) | Control  Low  High | BL  BL  BL | BL  BL  BL | BL  BL  BL | BL  BL  BL | BL  BL  BL |
| Cannabidiol (CBD) | Control  Low  High | BL  BL  BL | BL  BL  BL | BL  BL  BL | BL  BL  BL | BL  BL  BL |
| Cannabigerolic Acid (CBGA) | Control  Low  High | 0.1746 ± 0.0127  0.1243 ± 0.0096  0.1645 ± 0.0241 | 0.2991 ± 0.0266  0.2471 ± 0.0338  0.2440 ± 0.0236 | 0.2423 ± 0.0111  0.1788 ± 0.0158  0.2109 ± 0.0166 | 0.3907 ± 0.0325  0.3603 ± 0.0283  0.3891 ± 0.0291 | 0.6361 ± 0.0309  0.5702 ± 0.0545  0.5994 ± 0.0449 |
| Cannaabidivarin (CBDV) | Control  Low  High | ND  ND  ND | ND  ND  ND | ND  ND  ND | ND  ND  ND | ND  ND  ND |
| Cannabidiolic Acid (CBDA) | Control  Low  High | 1.9108 ± 0.0674  1.7743 ± 0.0818  2.0298 ± 0.1143 | 2.5343 ± 0.1312  2.1955 ± 0.1529  2.2185 ± 0.1299 | 3.2529 ± 0.1185  2.6308 ± 0.1443  2.8221 ± 0.1448 | 5.3494 ± 0.3191  4.5474 ± 0.3728  4.6047 ± 0.2747 | 7.6527 ± 0.4327  6.1125 ± 0.5196  6.5797 ± 0.3126 |
| Delta 9 Tetrahydro-cannabinolic Acid (THCA) | Control  Low  High | 0.0882 ± 0.0041  0.0800 ± 0.0049  0.0945 ± 0.0065 | 0.1281 ± 0.0080  0.1060 ± 0.0096  0.1060 ± 0.0075 | 0.1877 ± 0.0062  0.1496 ± 0.0080  0.1593 ± 0.0082 | 0.2260 ± 0.0163  0.2245 ± 0.0193  0.2337 ± 0.0145 | 0.3880 ± 0.0223  0.3156 ± 0.0275  0.3324 ± 0.0161 |
| Tetrahydrocannabidivarin (THCV) | Control  Low  High | ND  ND  ND | ND  ND  ND | ND  ND  ND | ND  ND  ND | ND  ND  ND |
| Delta 9 THC | Control  Low  High | BL  BL  BL | BL  BL  BL | BL  BL  BL | BL  BL  BL | BL  BL  BL |

ND = Not detected or no peak present

BL = Below the Limit of Quantitation but above the Limit of Detection

**Supplementary Table 3** Mean ± SEM percentage concentration by dry weight for all tested cannabinoids for 2021 var. Autoflower, infested with *T. urticae.* Total THC/CBD concentration used in Chapter 1 were calculated using THC = THCA x 0.877 + Delta 9 THC; CBD = CBDA x 0.877 + CBD.

| Cannabinoid | Infestation  Level | Days After Infestation (DAI) | | |
| --- | --- | --- | --- | --- |
|  |  | Pre infestation | 7 DAI | 14 DAI |
| Cannabinol (CBN) | Control  Low | ND  ND | ND  ND | ND  ND |
| Delta 8 THC | Control  Low | ND  ND | ND  ND | ND  ND |
| Cannabichromene (CBC) | Control  Low | ND  ND | ND  ND | ND  ND |
| Cannabigerol (CBG) | Control  Low | ND  ND | ND  ND | ND  ND |
| Cannabidiol (CBD) | Control  Low | BL  BL | BL  BL | BL  BL |
| Cannabigerolic Acid (CBGA) | Control  Low | 0.0567 ± 0.0103  0.0525 ± 0.0056 | 0.0856 ± 0.0110  0.1762 ± 0.0396 | 0.0950 ± 0.0110  0.1318 ± 0.0396 |
| Cannaabidivarin (CBDV) | Control  Low | ND  ND | ND  ND | ND  ND |
| Cannabidiolic Acid (CBDA) | Control  Low | 2.4012 ± 0.2906  2.6165 ± 0.2076 | 1.8353 ± 0.2381  2.6277 ± 0.5288 | 5.6270 ± 0.9521  7.4850 ± 0.8450 |
| Delta 9 Tetrahydro-cannabinolic Acid (THCA) | Control  Low | 0.1106 ± 0.0126  0.1169 ± 0.0104 | 0.1106 ± 0.0151  0.1551 ± 0.0299 | 0.2072 ± 0.0337  0.2753 ± 0.0299 |
| Tetrahydrocannabidivarin (THCV) | Control  Low | ND  ND | ND  ND | ND  ND |
| Delta 9 THC | Control  Low | BL  BL | BL  BL | BL  BL |

ND = Not detected or no peak present

BL = Below the Limit of Quantitation but above the Limit of Detection
